# Correction: Dastghaib et al. Simvastatin Induces Unfolded Protein Response and Enhances Temozolomide-Induced Cell Death in Glioblastoma Cells. *Cells* 2020, *9*, 2339

**DOI:** 10.3390/cells13080722

**Published:** 2024-04-22

**Authors:** Sanaz Dastghaib, Shahla Shojaei, Zohreh Mostafavi-Pour, Pawan Sharma, John B. Patterson, Afshin Samali, Pooneh Mokarram, Saeid Ghavami

**Affiliations:** 1 Department of Biochemistry, School of Medicine, Shiraz University of Medical Sciences, Shiraz 7134845794, Iran; suny.respina@gmail.com (S.D.); zmostafavipour88@yahoo.co.uk (Z.M.-P.); 2 Endocrinology and Metabolism Research Center, Nemazee Hospital, Shiraz University of Medical Sciences, Shiraz 7193635899, Iran; 3 Department of Human Anatomy and Cell Science, Rady Faculty of Health Sciences, Max Rady College of Medicine, University of Manitoba, Winnipeg, MB R3E 0J9, Canada; Shahla.Shojaei@umanitoba.ca; 4 Maternal-Fetal Medicine Research Center, School of Medicine, Shiraz University of Medical Sciences, Shiraz 7134845794, Iran; 5 Center for Translational Medicine, Division of Pulmonary, Allergy and Critical Care Medicine, Jane & Leonard Korman Respiratory Institute, Sidney Kimmel Medical College, Thomas Jefferson University, Philadelphia, PA 19107, USA; pawan.sharma@jefferson.edu; 6 Orinove, Newbury Park, CA 91320, USA; John.Patterson@orinove.com; 7 Apoptosis Research Centre, National University of Ireland, H91 W2TY Galway, Ireland; afshin.samali@nuigalway.ie; 8 Autophagy Research Center, Shiraz University of Medical Sciences, Shiraz 7134845794, Iran; 9 Colorectal Research Center, Shiraz University of Medical Sciences, Shiraz 7193635899, Iran; 10 Faculty of Medicine, Katowice School of Technology, 40-555 Katowice, Poland

In the original publication [1], there was a mistake in Figures 4A, 6A, 7A and 9A as published. The protein loading controls (GAPDH) were the same. The corrected Figure 4A, Figure 6A, Figure 7A and Figure 9A appear below. The authors state that the scientific conclusions are unaffected. This correction was approved by the Academic Editor. The original publication has also been updated.

## Figures and Tables

**Figure 4:**
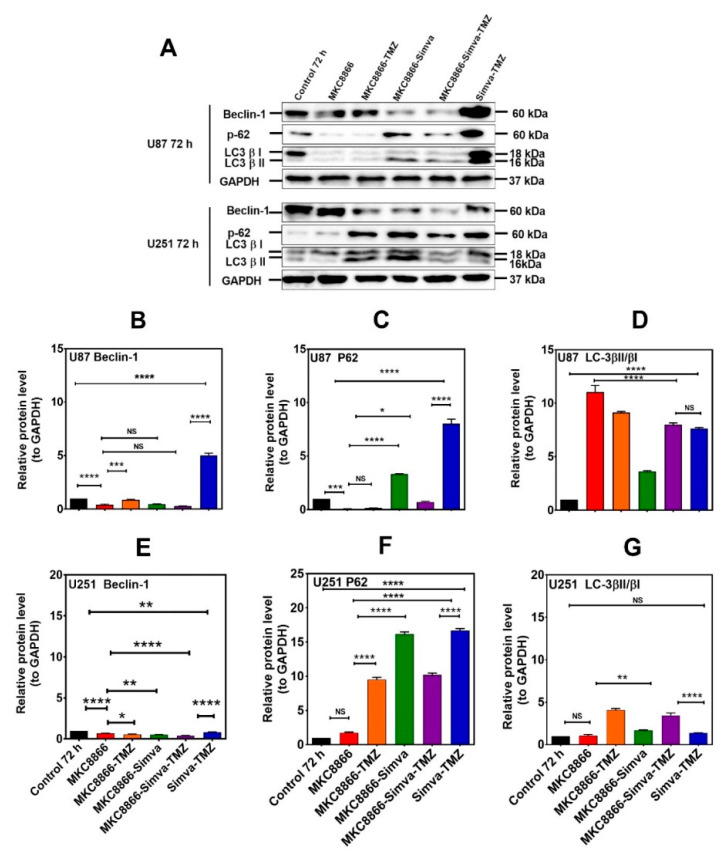
Simva–TMZ modulates the autophagy machinery via the IRE-1 pathway. (**A**) After pretreatment with MKC8866 (30 µM, 4 h), U87 and U251 cells were co-treated with TMZ, Simva, or Simva–TMZ for 72 h. The protein levels of Beclin-1, p62, LC3β-II, and LCβ-I were determined by immunoblotting. Simva–TMZ induced an inhibition of autophagy flux (accumulation of p62 and LC3β-II) in GBM cells. In Simva–TMZ-treated cells, MKC8866 increased p62 and Beclin-1 degradation, while it differentially affected the LC3β-II/LC3β-I ratio; GAPDH was used as loading control. Densitometric analysis of the Western blot bands confirmed that Simva–TMZ significantly induced Beclin-1 and p62 accumulation in both U87 and U251 cells (*p* < 0.0001), which was markedly prevented in the presence of MKC8866 (**B**,**C**,**E**,**F**). In addition, MKC8866 increased the LC3β-II/LC3β-I ratio in Simva–TMZ-treated U251 cells (*p* < 0.0001) (**G**), whereas it did not change LC3β-II/LC3β-I in U87 cells (*p* < 0.0001) (**D**). The data are shown as the mean ± SD from three independent experiments (* *p* < 0.05; ** *p* < 0.01, *** *p* < 0.001; **** *p* < 0.0001).

**Figure 6:**
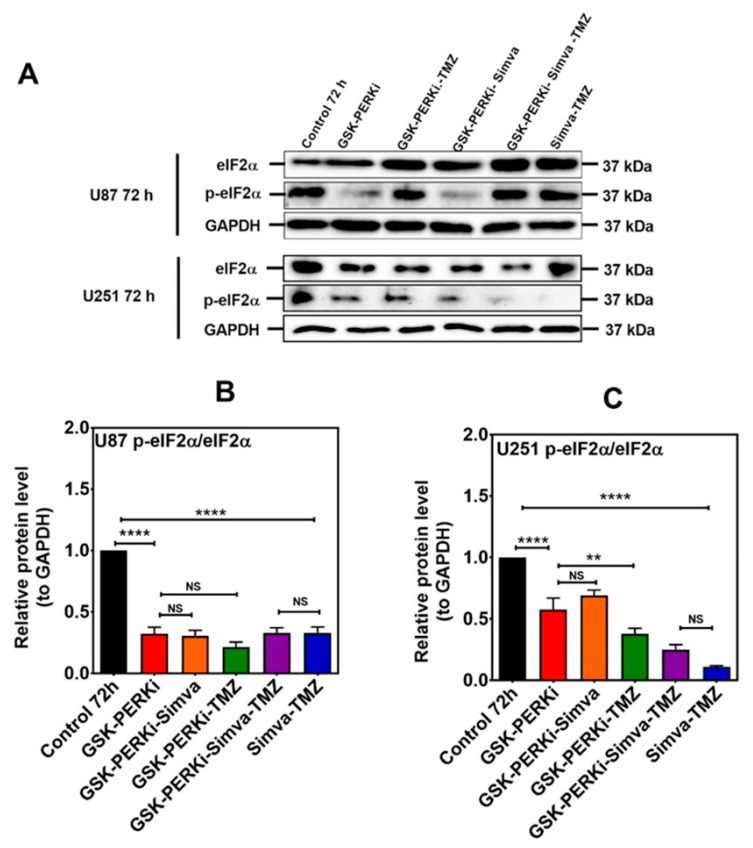
PERK inhibition does not change the p-eIF2α/eIF2α ratio in Simva–TMZ-treated cells. (**A**) U87 and U251 were pretreated with PERKi (5 µM, 30 min) and then co-treated with Simva–TMZ for 72 h. The protein levels of eIF2α and p-eIF2α were determined using immunoblotting; GAPDH was used as a loading control. (**B**,**C**) Densitometric analysis of the immunoblots showed that Simva–TMZ by itself significantly reduced the p-eIF2α/eIF2α ratio, which was not further decreased by the PERKi in either cell line. Of note, control levels of p-eIF2α were significantly decreased by the PERKi as well. The data are expressed as the means ± SD of three independent experiments ** *p* < 0.01; **** *p* < 0.0001).

**Figure 7:**
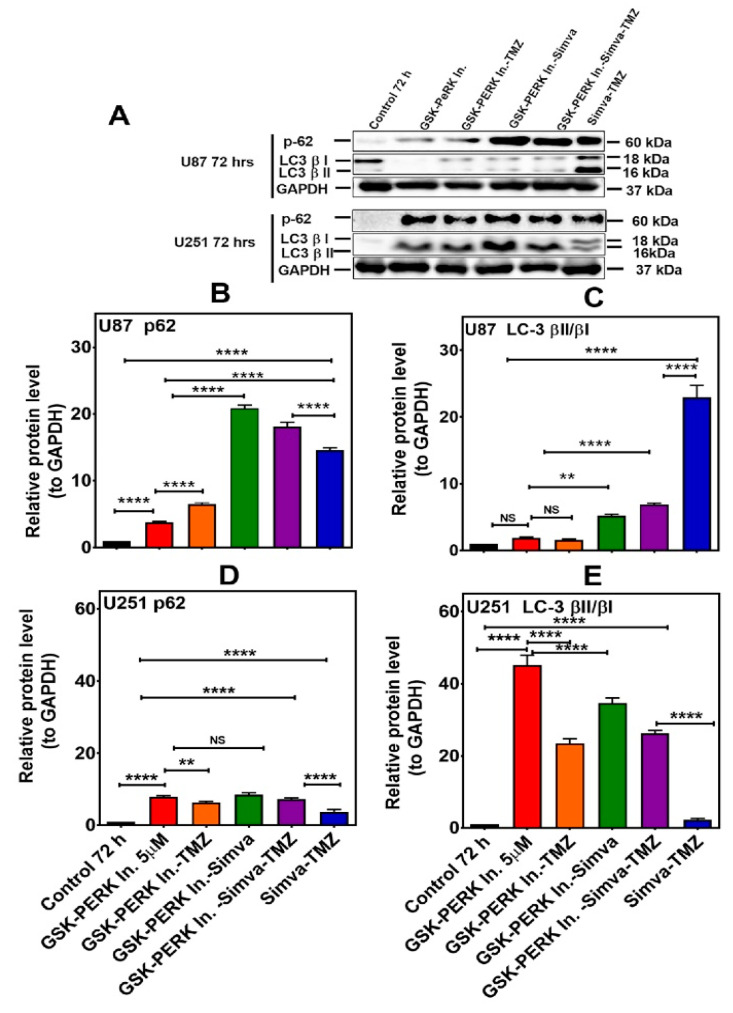
PERK inhibition differentially affects autophagy flux in U87 and U251 cells treated with Simva–TMZ. (**A**) U87 and U251 cells were pretreated GSK PERK inhibitor (5 µM, 30 min) and then co-treated with Simva–TMZ as described for 72 h. The protein levels of p62, LC3β-II, and LCβ-I were determined by immunoblotting. Simva–TMZ induced an inhibition of autophagy flux (accumulation of p62 and LC3β-II) in GBM cells. The PERKi decreased p62 degradation (autophagosome degradation) in both U87 and U251 cells, while it increased the LC3β-II/LC3β-I ratio in U251 cells and decreased it in U87 cells. GAPDH was used as a loading control. (**B**–**E**) Densitometric analysis of the Western blot bands to quantify p62 and LC3β-II/LC3β-I protein amount. Data are expressed as the mean ± SD of three independent experiments (** *p* < 0.01; **** *p* < 0.0001).

**Figure 9:**
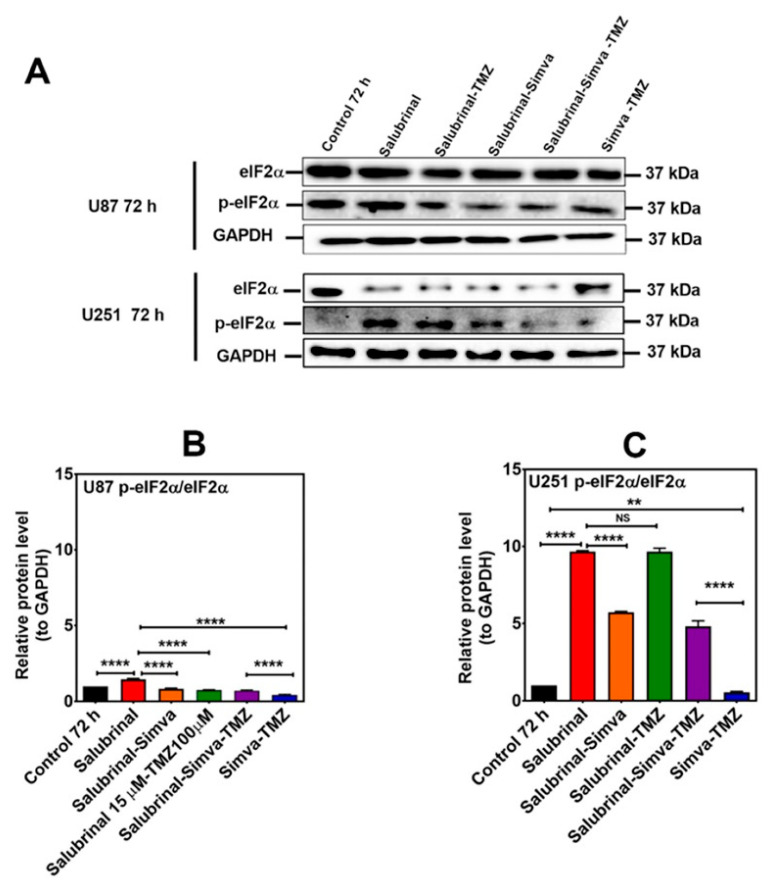
p-eIF2α phosphatase inhibition increases the p-eIF2α/eIF2α ratio in Simva–TMZ treated in GBM cells. (**A**) U87 and U251 cells were pretreated with salubrinal (15 µM, 30 min) followed by co-treatment with Simva–TMZ for 72 h. Cell lysates were collected, and the p-eIF2α/eIF2α protein amount ratios were determined using immunoblotting; GAPDH was used as a loading control. (**B**,**C**) Densitometric analysis of the Western blot bands shows that salubrinal significantly (*p* < 0.0001) increased the p-eIF2α/eIF2α ratio with Simva–TMZ treatment. Data are expressed as the means ± SD of three independent experiments (** *p* < 0.01, **** *p* < 0.0001).
