# Supplementary material for: Simvastatin Induces Unfolded Protein Response and Enhances Temozolomide-Induced Cell Death in Glioblastoma Cells
Source: Cells. 2020 Oct 22;9(11):2339. doi: 10.3390/cells9112339 (PMC7690447; doi:10.3390/cells9112339)

Simvastatin Induces Unfolded Protein Response and Enhances Temozolomide-induced Cell Death in Glioblastoma Cells

Sanaz Dastghaib[2,3], Shahla Shojaei[4], Zohreh Mostafavi-Pour[2,5], Pawan Sharma[6], John B. Patterson[7], Afshin Samali[8], *Pooneh Mokarram[1,2,9], Saeid Ghavami[1,4,10*]

Autophagy Research Center, Shiraz University of Medical Sciences, Shiraz, 7134845794, Iran
[2] Department of Biochemistry, School of Medicine, Shiraz University of Medical Sciences, Shiraz, 7134845794 Iran
[3] Endocrinology and Metabolism Research Center, Nemazee Hospital, Shiraz University of Medical Sciences, Shiraz, 7193635899 , Iran
[4] Department of Human Anatomy and Cell Science, Rady Faculty of Health Sciences, Max Rady College of Medicine, University of Manitoba; Winnipeg R3E 0J9, Canada; Shahla.Shojaei@umanitoba.ca
[5] Maternal-Fetal Medicine Research Center, School of Medicine, Shiraz University of Medical Sciences, Shiraz, 7134845794 , Iran
[6] Center for Translational Medicine, Division of Pulmonary, Allergy and Critical Care Medicine, Jane & Leonard Korman Respiratory Institute, Sidney Kimmel Medical College, Thomas Jefferson University, Philadelphia, PA 19107, USA; pawan.sharma@jefferson.edu
[7] Orinove, Newbury Park, California, 91320, USA, John.Patterson@orinove.com
[8] Apoptosis Research Centre, National University of Ireland, Galway, H91 W2TY, Ireland; afshin.samali@nuigalway.ie
[9] Colorectal Research Center, Shiraz University of Medical Sciences, Shiraz, 7193635899, Iran
[10] Faculty of Medicine, Katowice School of Technology, Katowice, 40-555, Poland
* Correspondence: mokaram2@gmail.com or mokaramp@sums.ac.ir (P.M.), saeid.ghavami@umanitoba.ca (S.G.), TeL./Fax: +98-711-2303029 (P.M.), +1-(204)272-3061 (S.G.)

***Corresponding authors**: Saeid Ghavami, Department of Human Anatomy and Cell Science, Rady Faculty of Health Sciences, Max Rady College of Medicine, University of Manitoba; Winnipeg, Canada. Phone (Office): +1-(204)272-3061 Phone (Lab): +1-(204)-272-3071 Email: saeid.ghavami@umanitoba.ca. Pooneh Mokarram, Department of Biochemistry Shiraz University of Medical Sciences Shiraz, Iran Phone: Tel:+98-711-2303029, Cell phone: +98-917-7160754. Fax: +98-711-2303029. Email: mokaram2@gmail.com, mokaramp@sums.ac.ir

Supplementary Figure Legends:

Figure S1: Cytotoxic effects of mevalonate (MEV), temozolomide (TMZ) and simvastatin (Simva) in GBM cells. U87 and U251 cells were treated with different concentrations of **(A, B)** mevalonate (1- 500 mM), **(C, D)** TMZ (25--1000 μM), and **(E, F)** Simva (1-20 μM), after which cell viability was assessed by MTT assays at different time points (24–96 h). Control samples were treated with the solvent (DMSO for Simva and TMZ or methanol for MEV). Data are shown as mean ± SD of 15 replicates from three independent experiments (*P<0.05; **P<0.01, ***P<0.001, **** =P<0.0001).

Figure S2: Mevalonate does not prevent Simva-TMZ-induced cell death in GBM cell lines. U87 and U251 cells were pretreated with MEV (2.5 mM, 3 h) then co-treated with Simva, TMZ, and Simva-TMZ for 72 h. Control samples were treated with solvent (DMSO for Simva and TMZ or methanol for MEV). MTT assaywas performed to evaluate cell viability **(A, B)**. Simva and TMZ co-treatment significantly decreased cell viability as compared to control, Simva and TMZ treatment alone in both cell lines. As anticipated, MEV fully prevented the effects of Simva on cell viability, but it failed to affect Simva-TMZ-mediated cytotoxicity. Data are shown as mean ± SD of 15 replicates from three independent experiments (*P<0.05; **P<0.01, ***P<0.001, **** P<0.0001).

Figure S3: Mevalonate partially inhibits Simva-TMZ-induced cell death in GBM cells. After pre-treatment with MEV (2.5 mM, 3 h), U87 and U251 cells were co-treated with Simva, TMZ, and Simva-TMZ for 72 h. Cell Death was determined by the propidium iodide Nicoletti assay. Simva-TMZ co-treatment significantly increased apoptosis compared to the corresponding control, Simva, and TMZ treatment alone **(A)**. MEV almost completely inhibited Simva-induced apoptosis and partially prevented Simva-TMZ-induced apoptosis in h GBM cells **(B, C).** Data

are representative of three independent experiments and presented as mean ±SD (*P < 0.05; **P < 0.01, ***P<0.001).

Figure S4: Simva and TMZ co-treatment induces autophagy flux inhibition in GBM cells. (A) U87 and U251 cells were treated, with Simva, TMZ, or Simva-TMZ for 72 h. Cells were edlysedand proteins were extracted. Autophagy-related proteins (Beclin-1, p62, LC3β) were detected using immunoblotting. Simva-TMZ co-treatment inhibited the autophagic flux (as indicated by p62 accumulation and an increase of LC3β-II/ LC3β-I ratio) in GBM cells. **(B-G)** Densitometric quantification of Beclin-1, p62, and LC3β protein levels from immunoblots in U87 and U251 cells (normalized to GAPDH as loading control). Simva-TMZ co-treatment decreased degradation of p62, and increased Beclin-1 expression and the LC3β-II/ LC3β-I ratio in GBM cells. The data are shown as the mean ± SD from three independent experiments (*P<0.05; **P<0.01, ***P<0.001; ****P<0.0001).

Figure S5: Simva-TMZ-induced UPR in U87 cells. Densitometric analysis of the immunoblot bands of various protein markers of UPR (GRP-78, IRE-1, XBP-1s, ATF6, and p-eIF2αeIF2α ratio) in U87 cells (normalized to GAPDH as loading control.) Simva –TMZ significantly induced UPR, and apoptosis in these cells (P<0.0001). The data are shown as the mean ± SD from three independent experiments (*P<0.05; **P<0.01, ***P<0.001; ****P<0.0001).

Figure S6: Simva-TMZ-induced UPR in U251 cells. Densitometric analysis of the immunoblot bands of various protein markers of UPR (GRP-78, IRE-1, XBP-1s, ATF6, and p-eIF2α/eIF2α ratio) in U251 cells (normalized to GAPDH as loading control). Simva–TMZ significantly induced UPR, and apoptosis in these cells (P<0.0001). The data are shown as the mean ± SD from three independent experiments (*P<0.05; **P<0.01, ***P<0.001; ****P<0.0001).

Supplementary Figure 1

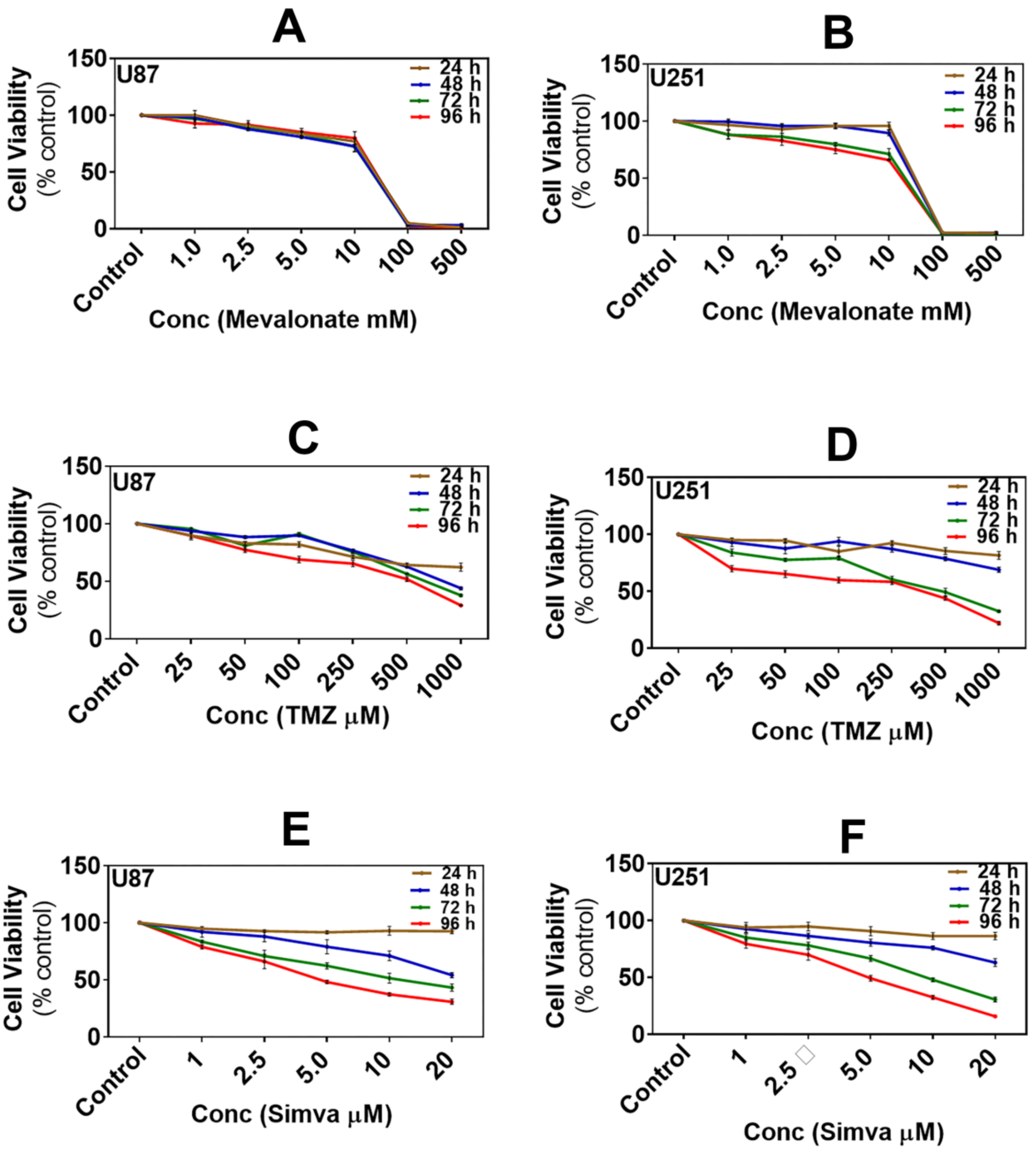

Supplementary Figure 2

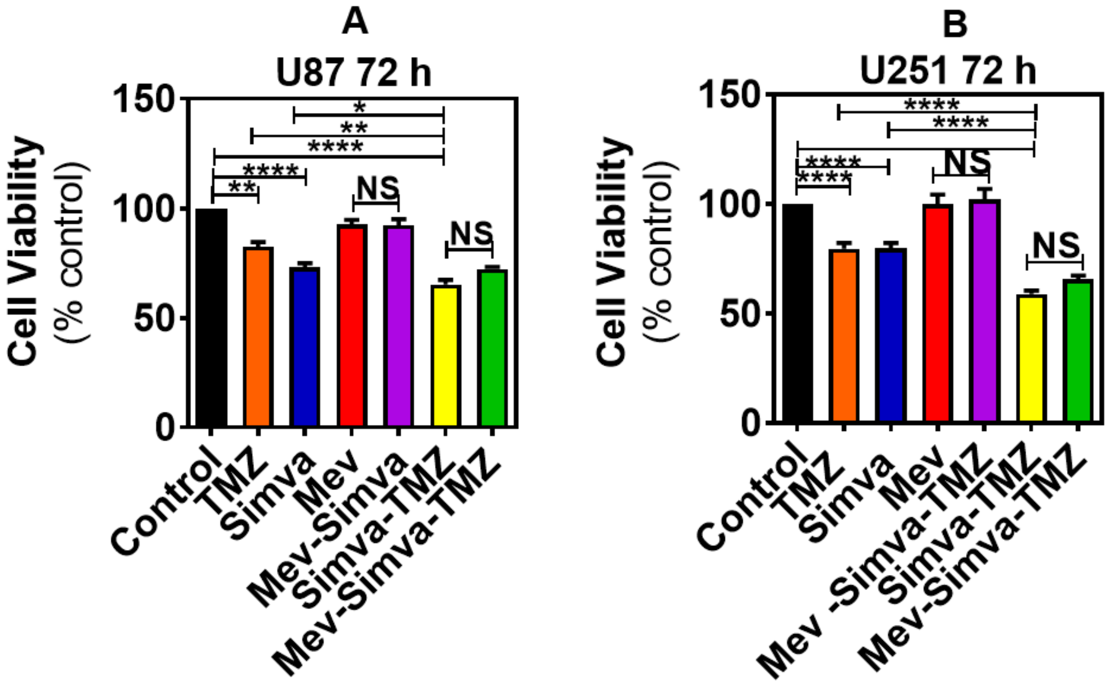

Supplementary Figure 3

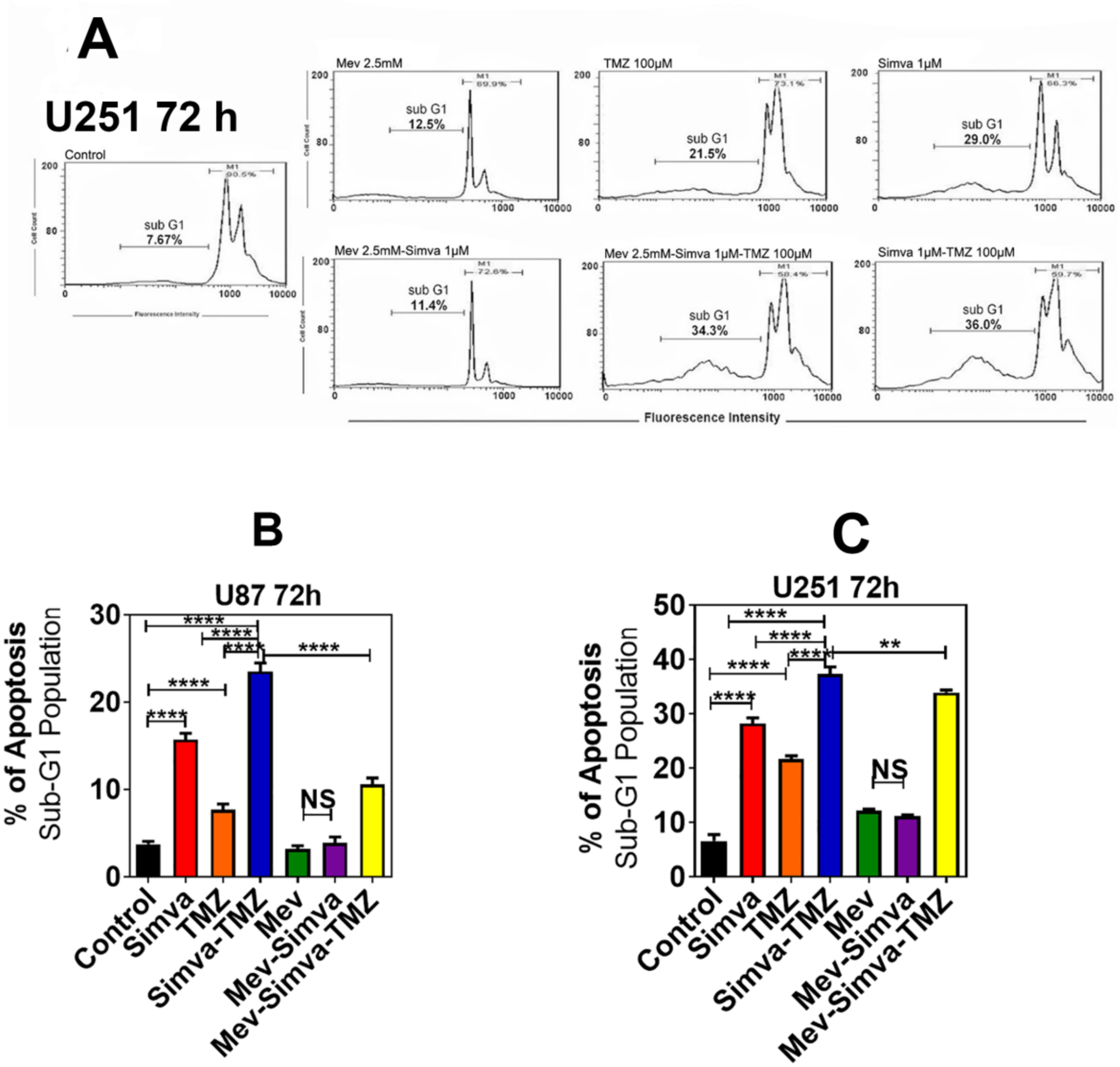

Supplementary Figure 4

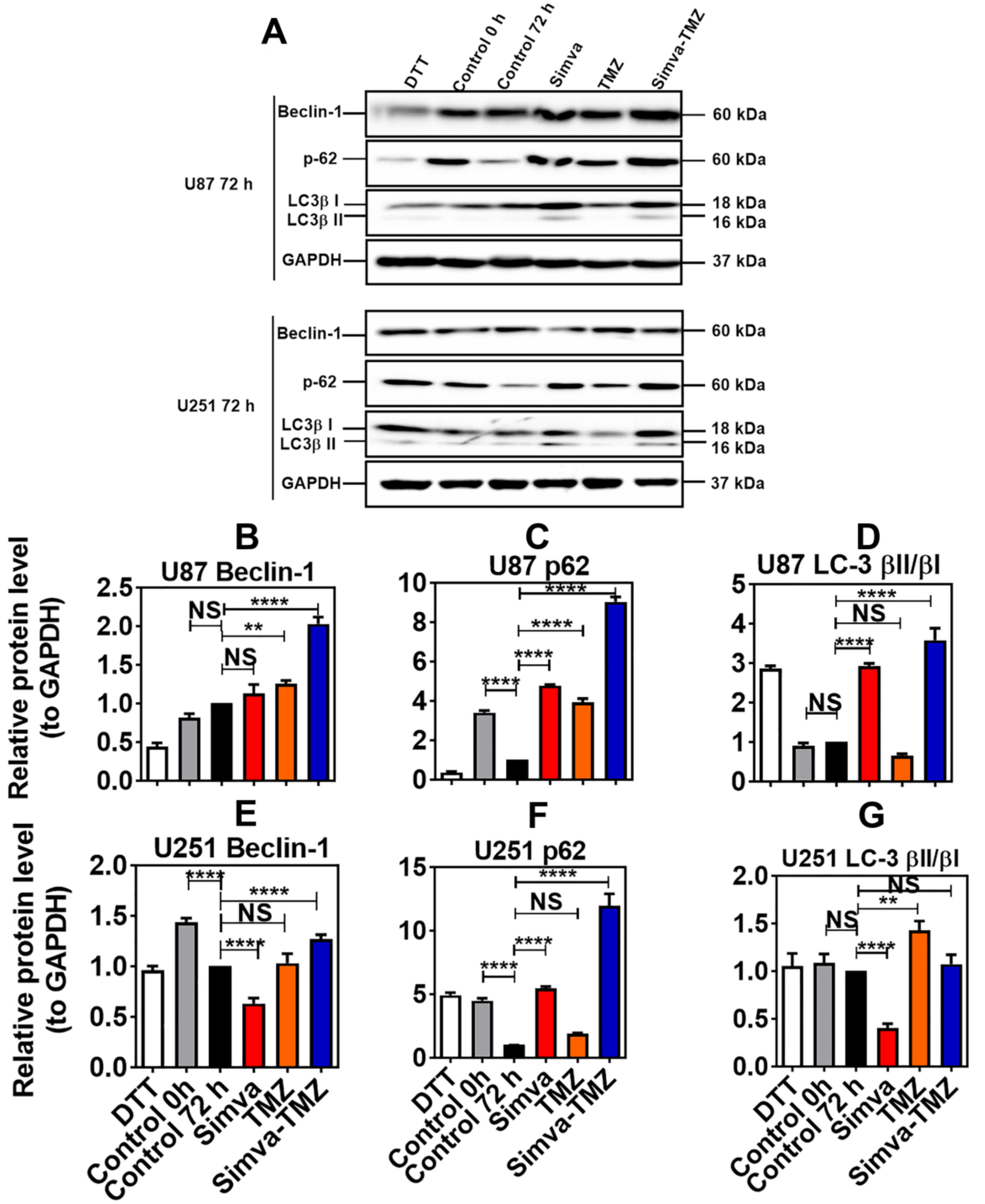

Supplementary Figure 5

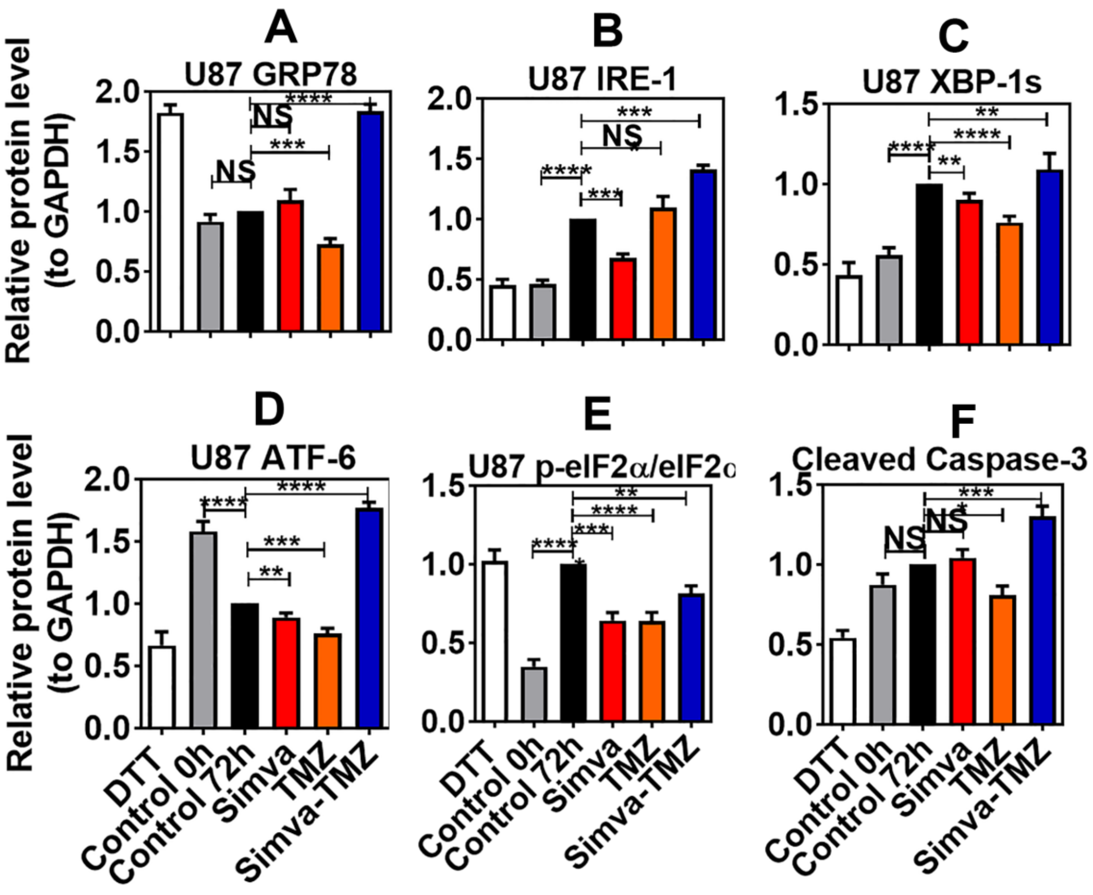

Supplementary Figure 6

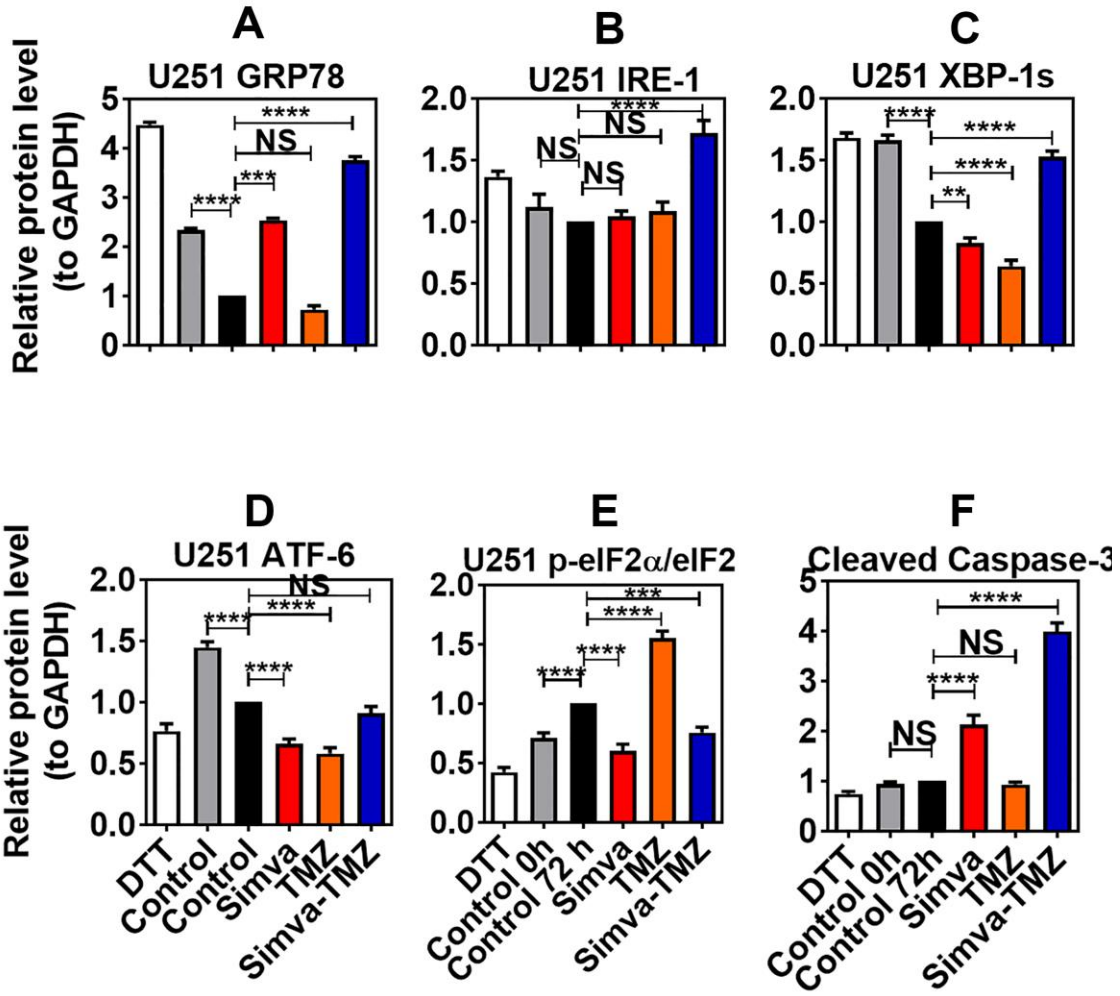

Supplement: Supplementary file 1 [file cells-09-02339-s001.pdf]
